# Enhanced Organic Contaminant Retardation by CTMAB-Modified Bentonite Backfill in Cut-Off Walls: Laboratory Test and Numerical Investigation

**DOI:** 10.3390/ma16031255

**Published:** 2023-02-01

**Authors:** Haijie He, Tao Wu, Xiaole Shu, Kuan Chai, Zhanhong Qiu, Shifang Wang, Jun Yao

**Affiliations:** 1 College of Civil and Architectural Engineering, Taizhou University, Taizhou 318000, China; 2 College of Civil and Architectural Engineering, Zhejiang University, Hangzhou 310000, China; 3 Fangyuan Construction Group Co., Ltd., Taizhou 317700, China; 4 College of Civil Engineering and Architecture, Jiangsu University of Science and Technology, Zhenjiang 212000, China; 5 MOE Key Laboratory of Soft Soils and Geoenvironmental Engineering, Zhejiang University, Hangzhou 310000, China; 6 College of Civil and Architectural Engineering, Shenyang Jianzhu University, Shenyang 110168, China; 7 China School of Mechanics and Civil Engineering, China University of Mining and Technology, Xuzhou 221018, China

**Keywords:** organic pollutants, organic bentonite, soil column, numerical simulation, kaolin

## Abstract

Adding organically modified bentonite into impervious wall materials may improve the adsorption of organic pollutants. In this study, cetyltrimethylammonium bromide organically modified bentonite (CTMAB bentonite) was mixed with sodium bentonite and kaolin to obtain two materials, which were then used as cut-off walls for typical pollutants. Soil column consolidation tests, diffusion tests, and breakdown tests were conducted to study migration of organic pollutants in soil columns. The parameter sensitivity of pollutant transport in the cut-off wall was analysed by numerical simulation. The sodium bentonite mixed with 10% CTMAB bentonite and kaolin-CTMAB bentonite showed the greatest impermeability: with a consolidation pressure of 200 kPa, the permeability coefficients were 1.03 × 10^−8^ m/s and 3.49 × 10^−9^ m/s, respectively. The quantity of phenol adsorbed on sodium bentonite-CTMAB bentonite increased with increasing water head height. The kaolin-CTMAB bentonite column showed the best rhodamine B adsorption performance, and the adsorption rate reached 98.9% on day 67. The numerical results showed that the permeability coefficient was positively correlated with the diffusion of pollutants in the soil column. The quantity adsorbed on the soil column was positively correlated with the retardation factor, and the extent of pollutant diffusion was negatively correlated with the retardation factor. This study provides a technical means for the optimal design of organic pollutant cut-off walls.

## 1. Introduction

Organic pollutants occupy a large proportion of highly polluted sites, and organic pollutants are commonly toxic, persistent, and volatile. Two organic pollutants are very typical: phenol is a derivative of benzene and a typical volatile organic compound, and rhodamine B is a synthetic cationic basic dye. Phenolic compounds are among the most common organic pollutants and exist widely in industrial wastewater discharged by the pharmaceutical, petrochemical, paper making, and machinery manufacturing industries [1]. Phenol is a refractory organic pollutant that persists in the environment and poses a long-term challenge to environmental safety. The United States Environmental Protection Agency (EPA) requires that the phenol content in wastewater cannot exceed 1 mg/L, and China’s Standards for Drinking Water Quality (GB5749-2006) stipulates an allowable limit for phenol in the water of 0.002 mg/L. Rhodamine B is one of the most toxic dyes found in dye wastewater; it can stimulate external mucous membranes, such as the eyes and skin, and cause diseases related to the digestive system and respiratory systems [2]. As a common dye, rhodamine B was once added to food, but in 1987, the International Agency for Research on Cancer (IARC) classified rhodamine B as a Class 3 carcinogen [3] and identified it as a threat to human health.

To prevent organic pollutants from entering the natural environment, cut-off walls are often used to complete in situ isolation. Among various factors affecting pollutants, adsorption is an important part of the barrier effect of cut-off wall materials [4,5]. To effectively remove pollutants from wastewater, scholars have developed a variety of novel and efficient adsorbents [6,7,8]. Since the end of the last century, soil-bentonite cut-off walls have been used for the isolation and restoration of contaminated sites based on considerations of economy and effectiveness [9,10]. However, the traditional soil-bentonite material has many shortcomings, such as low adsorption efficiency, poor chemical compatibility, and high permeability caused by dry cracking. This material fails to meet the needs for the isolation of complex pollutants. To overcome the defects of this material, many scholars have developed organically modified bentonite and found that adding organically modified bentonite to the cut-off wall material provides better barrier effects and more effective removal of organic pollutants [4,5]. Cetyltrimethylammonium bromide (CTMAB) is a modifier used to improve the adsorption and impermeability of bentonite. Studies have shown that CTMAB-modified bentonite effectively adsorbs heavy metals, organic pollutants, and organic dyes from water [11,12,13]. Kaolin is mainly composed of kaolinite, illite, and a small amount of montmorillonite and is a new and inexpensive adsorptive material. Previous studies have proven that kaolin is effective for handling pollutants, but its desorption capacity should not be underestimated. According to previous studies, the desorption capacity of kaolin is 5–22.5 times that of loess [14], so it is risky to use kaolin as an independent adsorbent. Note that the combination of kaolin and bentonite exhibits good cation exchange capacity [15], and the exchange capacity is related to the dosages of kaolin and bentonite; cation exchange capacity is an important factor affecting adsorption. Adding organically modified bentonite to kaolin improves the antifouling performance of cut-off walls, which is of great significance for practical pollution treatment.

The current research focusing on cut-off walls is mainly based on their impermeability and adsorption capability, and research methods comprise field tests and laboratory tests. However, the proportions of cut-off wall materials and specific adsorption of individual pollutants are discussed less frequently. It is worth mentioning that adjusting the proportions of cut-off wall materials can improve the economic benefits of pollution control, and it is thus important to explore the specific adsorption of pollutants in complex environments is significant.

In addition, the study of organic pollutant migration inside cut-off walls provides theoretical guidance for separating pollutants with these walls. Considerable progress has been made in investigations of one-dimensional and two-dimensional organic pollutant migration and its effects on the cut-off wall. Researchers have used numerical simulations to study the influence of migration parameters on the breakdown times of pollutants and adsorption of pollutants on cut-off walls [16,17], but the migration of organic pollutants inside cut-off walls is not well understood. Comsol Multiphysics analytical software can analyse organic pollutant migration in cut-off walls under various working conditions with multiple physical fields, and the underground flow module or mathematical partial differential equation module can be used for simulation studies. For example, Alkizwini [18] used Comsol to simulate the migration of dye pollutants inside an organic kaolin cut-off wall to determine the diffusion of pollutants in the equilibrium state. Dagher et al. [19] used Comsol and developed a diffusion model for pollutants in engineered barriers that considered the influencing factors of the gas phase and liquid phase. Among the existing research on pollutant migration, there are relatively few studies on the influence of different factors on the migration of pollutants inside the cut-off wall. To further guide the restoration of increasingly strained land resources, it is necessary to study the migration of organic pollutants inside cut-off walls.

Here, to explore the migration of organic pollutants in these cut-off wall materials, migration parameters such as the hydraulic dispersion coefficient, permeability coefficient, and retardation factor in the soil column were determined with soil column consolidation tests, chloride ion diffusion tests, and organic pollutant migration breakdown tests. Then, based on these experiments, Comsol software was used to simulate organic pollutant migration in the soil column to verify the accuracy of the migration parameters obtained. Finally, the key parameters affecting the antifouling performance in the cut-off wall were studied by parameter sensitivity analysis.

## 2. Materials and Methods

### 2.1. Preparation of Test Materials

The sodium bentonite and kaolin used in this study were purchased from Hebei Lingshou Xingyuan Mineral Company (Lingshou, China). The sodium bentonite exhibited a cation exchange capacity of 31 cmol/kg, and the main components were montmorillonite (78.3%), calcium feldspar (14.6%), quartz (6.4%), and orthoclase (0.7%). The CTMAB, phenol, rhodamine B, 4-aminoantipyrine, potassium ferricyanide, ammonium chloride, and ammonia used in the tests were purchased from Shanghai Aladdin Biochemical Technology Co., Ltd. (Shanghai, China).

Organically modified bentonite was prepared by the wet method [20], and hereinafter, organically modified bentonite is called CTMAB bentonite. Preparation of CTMAB bentonite proceeded as follows: First, 100 g of sodium bentonite was weighed and put into a 1 L beaker, and 500 mL of deionized water was added to prepare a 20% slurry. Next, 25 g of CTMAB was weighed and added to the slurry, which was then stirred evenly. After shaking in a constant temperature shaker at 70 °C for 2 h, it was put into an electric air-drying oven at 70 °C overnight until cationic exchange between the surfactant and bentonite was complete. The prepared sample was removed, filtered, and rinsed, and silver nitrate solution was used to detect bromide ions. When no bromide ions were detected, the bentonite was put into an electric air-drying oven at 105 °C. After drying, the organic bentonite was removed for grinding and passed through a 100-mesh sieve. Thus, the preparation of CTMAB bentonite was completed.

### 2.2. Consolidation Tests

Consolidation tests were conducted as follows: 950 g, 900 g, and 850 g of sodium bentonite were evenly mixed with 50 g, 100 g, and 150 g of CTMAB bentonite, respectively, and 390 mL of deionized water was added to ensure that the moisture content was 39% and the soil sample was saturated. Thus, soil column samples with CTMAB bentonite contents of 5%, 10%, and 15% were obtained. Then, 100 g of sodium bentonite and 100 g of CTMAB bentonite were mixed with 900 g of kaolin, with 1 kg of kaolin as the control group. Later, 390 mL of deionized water was added to obtain three samples of kaolin, kaolin-sodium bentonite, and kaolin-CTMAB bentonite, all of which were saturated. Finally, quantitative soil samples were taken, and the initial density and relative density of each soil sample was determined and calculated according to Formulas (1) and (2).

(1) $$\rho_{0}=\frac{m_{0}}{V}$$

where *ρ*_0_ is the initial density of the sample (g/cm^3^); *m*_0_ is the sample mass (g); and *V* is the soil volume (cm^3^).

(2) $$G_{s}=\frac{m_{s}}{m_{w}}$$

where *m_w_* is the water mass at room temperature (g), and *m_s_* is the sample mass for the same volume as water at 4 °C (g).

After the measurements, the samples of the soil and water mixture were packed into a sample mould with an inner diameter of 10 cm and a height of 15 cm. First, porous stones and filter papers were put into the mould in turn, and the mixed soil sample was packed into the sample mould. Then, filter papers and porous stones were placed on top of the soil in turn, and the sample was compacted for 24 h under a weight of 1.275 kg. After compaction, consolidation tests were performed with homemade consolidation compressors.

The compression levels selected for the consolidation tests were 25 kPa, 50 kPa, 100 kPa, and 200 kPa, and the hourly deformations of the soil columns under each pressure level were recorded by an indexing metre. The measuring range of the indexing metre was 0–50 mm, and a compression change of less than 0.01 mm per hour was set as the stability criterion. When the reading of the indexing metre did not change, the pressure of this level was consolidated, and the next pressure level was applied. After the completion of pressure consolidation with 200 kPa, the loaded samples were removed. According to Formulas (3)–(8), the changes in the void ratio and permeability coefficient at each pressure level were calculated from changes in compression consolidation [14].

(3) $$e_{0}=\frac{\left(1+0.01w_{1}\right)G_{s}\rho_{w}}{\rho_{0}}-1$$

where *G_s_* is the relative density of the soil sample; *w*_1_ is the initial moisture content; *ρ*_0_ is the initial density of the soil sample (g/cm^3^); *ρ_w_* is the water density (taken as 1.0 g/cm^3^ here); and *e*_0_ is the initial void ratio of the soil sample.

(4) $$a_{v}=\frac{e_{i-1}-e_{i}}{p_{i}-p_{i-1}}$$

where *a_v_* is the compression coefficient (MPa^−1^); *p_i_* is the pressure value at each level; and the *a_v_* values of less compressible, moderately compressible, and highly compressible soil are *a_v_* < 0.1 MPa^−1^, 0.1 MPa^−1^ ≤ *a_v_* < 0.5 MPa^−1^, and *a_v_* ≥ 0.5 MPa^−1^, respectively.

(5) $$e_{i}=e_{0}-\frac{1+e_{0}}{h_{0}}\Delta h_{i}$$

where *h*_0_ is the initial height (mm); Δ*h_i_* is the height difference of the soil sample at each pressure level; and *e_i_* is the void ratio.

(6) $$C_{v}=\frac{0.848h^{2}}{t_{90}}$$

where *t*_90_ is the time required for the soil sample to reach 90% consolidation (s) and *h* is the maximum drainage distance, which was taken as 1/2 of the average initial and final heights of the soil samples under pressure (cm).

(7) $$E_{s}=\frac{1+e_{0}}{a_{v}}$$

where *e*_0_ is the natural void ratio of the sample and *a_v_* is the compression coefficient (MPa^−1^).

(8) $$k=\frac{C_{v}\gamma_{w}}{E_{s}}$$

where *γ_w_* is the unit weight of water, taken as 9.8 kg/m^3^ here.

Since dispersion is often accompanied by a difference in flow velocity, the dispersion coefficient is usually expressed as a function related to the seepage velocity, as shown in the following formula:

(9) $$D_{m}=\alpha v_{s}$$

where *α* is the dispersion coefficient, which is usually expressed by empirical formulas:

(10) $$\;\alpha =\frac{x^{2}}{100}m\;\;\;\;\;\;\;\;\;\;\;\;x\leq 100\;m\;\alpha =100m\;\;\;\;\;\;\;\;\;\;\;\;x>100\;m$$

where *x* is the dispersion scale of pollutants (*m*).

Generally, the dispersion coefficient and diffusion coefficient are considered the hydraulic dispersion coefficients [21]:

(11) $$D_{h}=D_{m}+D^{*}$$

where *D_m_* is the dispersion coefficient, D* is the diffusion coefficient, and *D_h_* is the hydraulic dispersion coefficient.

The values of retardation factors were measured by batch adsorption tests with rhodamine B and phenol on six soil column materials (according to the adsorption test standard GB21851-2008), and the specific formulas used for calculation are:

(12) $$\rho_{d}=\frac{\rho_{0}}{\left(1+w\right)\rho_{0}}$$

(13) $$R_{\mathrm{dL}}=1+\frac{\rho_{d}K_{L}Q_{L}}{n{\left(1+K_{L}q_{e}\right)}^{2}}$$

(14) $$R_{\mathrm{dF}}=1+\frac{\rho_{d}}{n}K_{F}{\mathrm{Nq}}_{e}^{N-1}$$

where *ρ_d_* is the dry density of the soil (g/cm^3^); *w* is the moisture content of the soil; *ρ*_0_ is the initial density of the soil; *R_d_* is the retardation factor, a dimensionless constant; *K_L_* is the Langmuir constant, which is the maximum adsorption quantity used in the Langmuir simulation (mg/g); *q_e_* is the adsorption quantity per unit (mg/g); *N* and *K_F_* are Freundlich constants; and *n* is the void ratio of the soil.

### 2.3. Chloride Ion Diffusion Tests

The six soil columns consolidated and compressed at 200 kPa were tested for chloride ion diffusion at a water head height of 150 cm. NaCl solution was selected as the diffusion solution, and the diffusion test lasted for five days. At the end of the diffusion process, the soil column was sliced, and the obtained soil column was equally divided into seven layers longitudinally and five layers transversely to obtain samples with different heights and radii. The samples were put into a 100 mL centrifuge tube, and 90 mL of deionized water was added. Then, the centrifuge tube was put into a shaking bath at 300 rpm for 2 h at room temperature. Subsequently, the chloride ions were eluted into the solution and centrifuged at 4000 rpm for 10 min. The supernatant was removed and tested by ion chromatography to obtain the ion concentrations for different heights and radii of the soil column. The apparent bending factor of the soil was calculated with Formula (15). The specific test scheme is described in Table 1.

(15) $$\frac{C}{C_{0}}=erfc\left(\frac{z}{2\sqrt{\tau D_{0}t}}\right)$$

where *z* is the longitudinal height of the soil column; *τ* is the bending factor of the soil column; *D*_0_ is the chloride ion diffusion coefficient; *C* is the chloride ion concentration in the soil column at different heights; *C*_0_ is the initial chloride ion concentration; and *t* is the diffusion time of chloride ions in the soil column.

### 2.4. Migration Breakdown Test of Organic Pollutants

**Figure 1:**
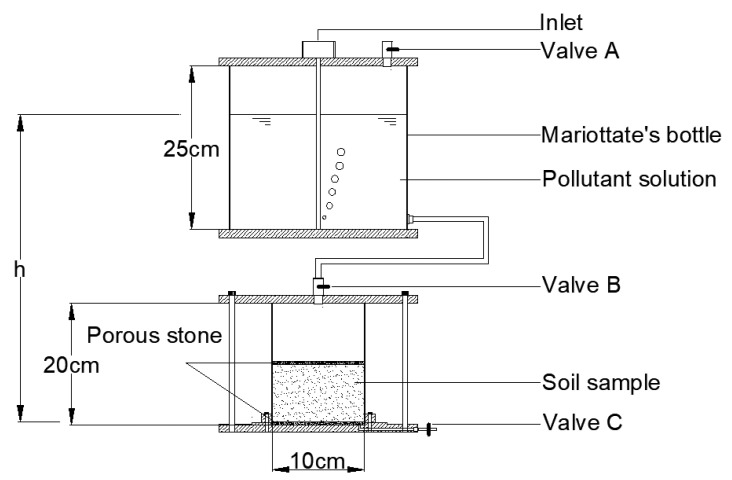
Migration breakdown test device.

Figure 1 shows the test device used in the migration breakdown tests. The detailed process was as follows: First, valve A was opened, valve B and valve C were closed, 150 mg/L phenol solution was poured into the inlet of the Mariotte bottle, and the water head was adjusted to a height *h*. After sample preparation in the lower part was completed, valves B and C were opened, valve A was closed, and the phenol solution in the Mariotte bottle was added into the soil column according to a certain flow rate. It is stipulated that the *h* values of different water head heights were 37.5, 75, 112.5, and 150 cm. Taking the 150 cm water head height as an example, two soil columns at each height were prepared for transverse and longitudinal tests. The test time was 20 days. After the test was completed, valves B and C were closed, the samples were removed by a demoulding machine, and each soil column sample was sliced into layers. Soil Column 1 was sliced and sampled at longitudinal heights of 2.66, 5.32, 7.98, 10.64, and 13.3 cm. Soil Column 2 was sliced and sampled with transverse radii of 2, 3, 4, and 5 cm. The sections were put into brown bottles, and the phenol content in each section was detected with gas chromatography-mass spectrometry (GC-MS) to determine the longitudinal and transverse migration of phenol in the soil column with different water head heights.

The prepared kaolin, kaolin-sodium bentonite, and kaolin-CTMAB bentonite columns were subjected to migration breakdown tests with 150 mg/L rhodamine B solution. Following the same steps as the migration breakdown test for phenol, the beaker was connected under the test mould, and the leachate was tested. With a 150 cm water head height, the testing times for the leachate were 10, 20, 35, 39, 41, 43, 50, and 67 days. The spectrophotometric method [22] was used to detect leachate and explore the change in adsorption of rhodamine B by the soil columns over time. The specific test scheme is shown in Table 1.

## 3. Results and Discussion

### 3.1. Void Ratio Variations of Soil Columns with Consolidation Pressure

**Figure 2:**
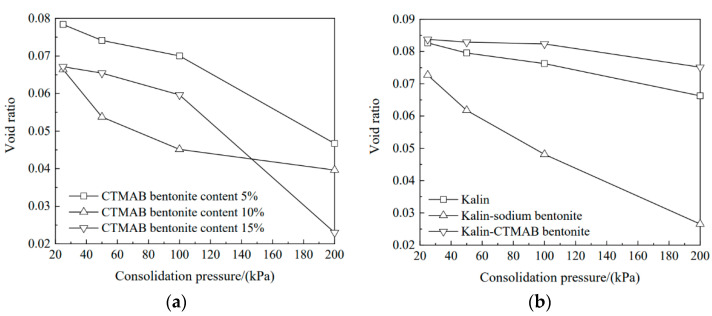
Void ratio variations of soil columns with increasing consolidation pressures: (**a**) sodium bentonite-CTMAB bentonite, (**b**) kaolin, kaolin-sodium bentonite, and kaolin-CTMAB bentonite.

As shown in Figure 2a, the void ratio gradually decreased with increasing consolidation pressure. At 25 kPa, with more CTMAB bentonite added, the void ratio decreased. When the consolidation pressure was increased from 25 kPa to 100 kPa, the soil column with 10% CTMAB bentonite content showed good compressibility. At 100 kPa, the soil column with 10% CTMAB bentonite content had the lowest void ratio among the three columns; the void ratios with pressures of 25 kPa, 50 kPa, and 100 kPa were 0.0700, 0.0451, and 0.0596, respectively. At 200 kPa, the soil column with 15% CTMAB bentonite content showed the largest change, and the void ratio decreased by 12% compared with that of the previous level (100 kPa). The final void ratios of the three soil columns were 0.0466, 0.0396, and 0.0229. These results showed that with increasing CTMAB bentonite content, void ratio variations for the soil columns showed a certain delay. This is because the addition of CTMAB bentonite enhanced the compressive strength of the soil column. The long carbon chain cation replaced Na ions from montmorillonite and improved the compressive strength of the montmorillonite structure. Overall, the void ratio gradually decreased with increasing CTMAB bentonite content.

Figure 2b shows that the soil column with sodium bentonite had the largest variation in the void ratio with increasing consolidation pressure, and the soil column with CTMAB bentonite showed the worst compressibility. In addition, the adsorbent with CTMAB bentonite exhibited the lowest compression: the modification inhibited the formation of a polymer between sodium bentonite and kaolin, and it replaced the Na ions between montmorillonite layers, made the interlayer structure denser, and enhanced the compressive strength. The void ratio of kaolin-sodium bentonite was the smallest at 200 kPa, and those of kaolin, kaolin-sodium bentonite, and kaolin-CTMAB bentonite were 0.0662, 0.0265, and 0.0751, respectively.

### 3.2. Permeability Coefficient Variations for Soil Columns with Consolidation Pressure

**Figure 3:**
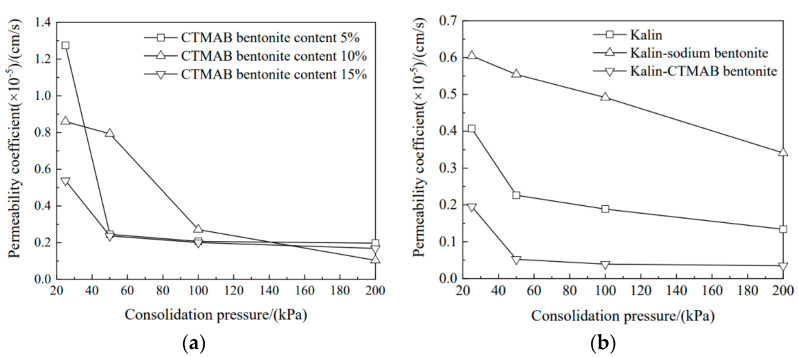
Permeability coefficient variations for soil columns with consolidation pressure: (**a**) sodium bentonite-CTMAB bentonite. (**b**) kaolin, kaolin-sodium bentonite, and kaolin-CTMAB bentonite.

As shown in Figure 3a, the permeability coefficient decreased at 25 kPa with increasing CTMAB bentonite content. With increasing consolidation pressure, the permeability coefficient decreased gradually. The permeability coefficient of the soil column with 5% CTMAB bentonite content decreased significantly at 50 kPa, from 1.27 × 10^−5^ cm/s to 2.45 × 10^−6^ cm/s, after which the consolidation pressure did not change significantly. Under a consolidation pressure of 200 kPa, the permeability coefficients of the three soil columns were 1.96 × 10^−6^, 1.03 × 10^−6^, and 1.68 × 10^−6^ cm/s.

As shown in Figure 3b, the soil column with sodium bentonite showed the best compressibility and the lowest void ratio with increasing consolidation pressure, and a consolidation pressure of 50 kPa decreased the permeability coefficients of kaolin and kaolin-CTMAB bentonite by 44% and 73%, respectively, compared with those at 25 kPa. Moreover, with increasing pressure, the permeability coefficients showed relatively small changes, and the permeability coefficients of kaolin and kaolin-CTMAB bentonite at 200 kPa were 1.33 × 10^−6^ and 3.49 × 10^−7^ cm/s, respectively. However, the permeability coefficient of the soil column with sodium bentonite was 3.41 × 10^−6^ cm/s at 200 kPa and was continuing to decrease at 200 kPa.

### 3.3. Diffusion of Chloride Ions in Different Soil Columns

Chloride ions are inert nonadsorbent ions, and the diffusion of chloride ions in soil column elements is determined by the bending barrier in the soil; the main index is expressed by the bending factor τ, which can be calculated with Formula (15) [23].

**Figure 4:**
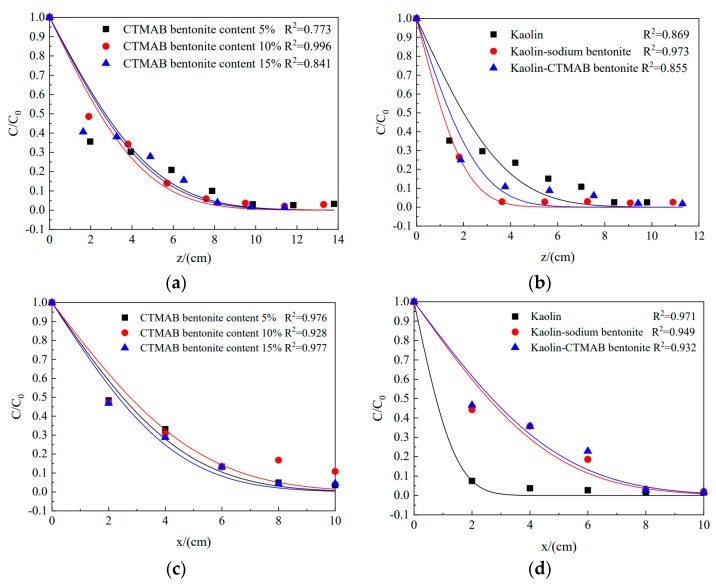
Concentration variations in diffusion tests: (**a**) longitudinal concentration variation of sodium bentonite-CTMAB bentonite; (**b**) longitudinal concentration variation of kaolin, kaolin-sodium bentonite, and kaolin-CTMAB bentonite; (**c**) transverse concentration variation of sodium bentonite-CTMAB bentonite; and (**d**) transverse concentration variation of kaolin, kaolin-sodium bentonite, and kaolin-CTMAB bentonite.

As seen from Figure 4a,c, the degrees of longitudinal and transverse diffusion by chloride ions in the three soil columns of sodium bentonite with 5%/10%/15% CTMAB were roughly the same, and the fitting curves were similar. The bending factors of the three soil columns were calculated as 0.7419, 0.7440, and 0.8510. With increasing CTMAB bentonite content, the bending factor of the soil also increased gradually. Figure 4b,d shows that in the longitudinal test, diffusion by chloride ions was greatest in the kaolin soil column, and the extent of diffusion was smallest in the kaolin-sodium bentonite soil column, indicating that the kaolin-bentonite polymer had a great influence on longitudinal diffusion. In the transverse test, the diffusion of chloride ions was smallest in the kaolin column, and the diffusion in the kaolin-sodium bentonite column and kaolin-CTMAB bentonite columns was approximately the same, which suggested that the granular nature of kaolin itself had a stronger retarding effect on the transverse diffusion of chloride ions. The specific diffusion coefficients are shown in Table 2.

### 3.4. Effect of Different Water Head Heights on Phenol Migration in the Soil Column

**Figure 5:**
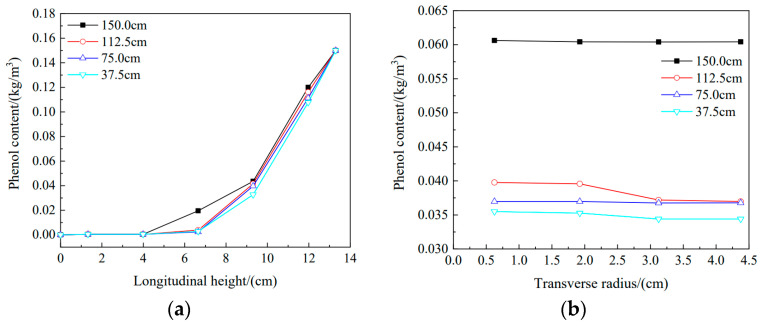
Variations in phenol levels in the soil column: (**a**) longitudinal phenol distribution, (**b**) transverse phenol distribution.

Figure 5a shows the results of longitudinal tests of the soil columns. From variations in phenol content at different soil column heights with water head height, the flow rate for phenol solution was accelerated with increasing water head height, the amount of phenol entering the soil column gradually increased, and migration of the phenol solution in the soil column gradually deepened. The first layer of the soil column has the highest phenol content, and the phenol contents measured at four heights were between 0.119 kg/m^3^ and 0.107 kg/m^3^. Taking the water head heights of 150.0 cm and 37.5 cm as examples, the concentrations of phenol in the upper three layers decreased by 10.0%, 25.0%, and 85.0%, respectively, with decreasing longitudinal height of the soil column, demonstrating that the decrease in the water head height caused a gradual decrease in the infiltration amount of phenol, and the two exhibited a positive relationship. Additionally, there was no phenol exudation in the bottom layer for the test with the highest head height, which indicated that the soil column has good adsorption capacity for phenol and could constitute a barrier to phenol.

Figure 5b shows the results of the transverse tests of the soil columns. The variations in phenol content for different soil column radii with water head height indicated that transverse diffusion of phenol solution in the soil column increased with increasing height. The phenol content decreased slightly with increasing soil column radius, and the effect was moderated by increasing water head height. At a water head height of 150 cm and a soil column radius of 4.375 cm, the phenol content was 0.0606 kg/m^3^, which was 0.3% lower than the phenol content of 0.0604 kg/m^3^ at a radius of 0.625 cm. At a water head height of 37.5 cm and a soil column radius of 4.375 cm, the phenol content was 0.0344 kg/m^3^, which was decreased by 3.0% compared with the phenol content of 0.0354 kg/m^3^ at a radius of 0.625 cm. This is because the higher the water head height is, the faster the dripping speed of the phenol solution. As time passed, the phenol solution formed a liquid layer on the upper surface of the soil column, thus reducing the transverse variation in phenol concentration.

### 3.5. Effect of Time on the Removal of Rhodamine B from Different Soil Columns

**Figure 6:**
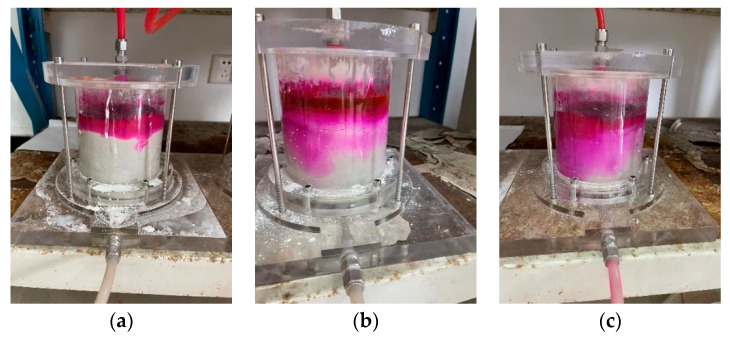
Variations in rhodamine B content in the soil column: (**a**) kaolin-CTMAB bentonite, (**b**) kaolin-sodium bentonite, and (**c**) kaolin.

**Figure 7:**
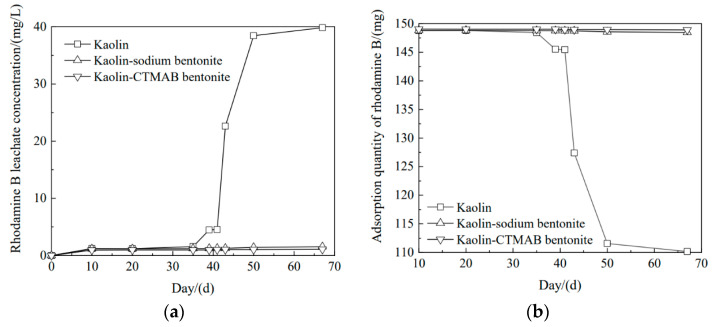
Variations in rhodamine B adsorption: (**a**) rhodamine B leachate concentration and (**b**) amount of rhodamine B adsorbed by the soil column.

Figure 6 shows the test results for the breakdown of the rhodamine B pollutant by the kaolin, kaolin-sodium bentonite, and kaolin-CTMAB bentonite columns. Shown from left to right are the adsorption diagrams for rhodamine B with kaolin, kaolin-sodium bentonite, and kaolin-CTMAB bentonite. Figure 7 shows the adsorption of rhodamine B over time. The kaolin column was broken down on the 35th day, the concentration of rhodamine B in the leachate gradually increased over the next 32 days, and the concentration was 39.8 mg/L on the 67th day. The total amount adsorbed by the soil column decreased from 148.7 mg to 110.1 mg with increasing time. Additionally, on Day 35, the leachate concentration of the kaolin-sodium bentonite column was 1.21 mg/L, the concentration of rhodamine B gradually increased from Day 35 to Day 67 and reached 1.52 mg/L on Day 67, and the total amount adsorbed decreased from 148.7 mg to 148.4 mg. In the same time period, the kaolin-CTMAB bentonite column showed the greatest adsorption of rhodamine B solution over 67 days. On Day 67, the concentration of leachate was 1.08 mg/L, and the total amount adsorbed was 148.9 mg. It was thus determined that the kaolin-CTMAB bentonite column adsorbed rhodamine B most effectively.

## 4. Numerical Simulation of Organic Pollutant Migration Breakdown

### 4.1. Mathematical Model and Governing Equation

Comsol software was used for simulation modelling. Geometric dimensions were set as half the width of the main viewing plane of the actual sodium bentonite-10% CTMAB bentonite column, with a transverse range of 0.0–5.0 cm and a longitudinal range of −1.0–13.3 cm. The right and bottom sections of the model are constraint conditions of 0 flux, initial conditions of 0 kg/m^3^ for internal concentration, and boundary conditions of 0.15 kg/m^3^ for the top surface. An infinite element domain with a thickness of 1 cm was added at the bottom of the model to avoid pollutant migration and accumulation caused by the constraint conditions at the bottom and to model the actual conditions as closely as possible.

The simulation results for phenol migration in the soil column were compared with the experimental results to verify the accuracy of the obtained data. The concentration boundary on the upper surface of the soil column was set as two stages due to the different flow rates caused by the different water head heights. The first stage was to set the concentration boundary at 0–0.5 cm, and the second stage was to set the concentration boundary at 0–5 cm. The variation in flow velocity for different water head heights was reflected by the different conversion times of the concentration boundary. The conversion times for the four heights were 24, 48, 72, and 120 h, respectively. The simulation results were obtained by importing migration parameters for four different heights. The settings for geometric models of the other three soil columns were similar to those described above. The longitudinal heights of the soil columns differed according to the actual situation, so the longitudinal ranges were different. For the kaolin soil column, the longitudinal range was −1.0–9.8 cm; for the kaolin-sodium bentonite soil column, the longitudinal range was −1.0–10.9 cm; and for the kaolin-CTMAB bentonite column, the longitudinal range was −1.0–11.3 cm. The migration parameters were imported to perform the simulations.

From the convection-diffusion equation, the governing equation was determined by considering migration caused by diffusion and diffusion effects in the transverse and longitudinal directions, migration caused by convection in the longitudinal direction, and the retarding effect of pollutant adsorption [24]; the resulting equation is as follows:

(16) $$R_{d}\frac{\partial C}{\partial t}=D_{x}\frac{\partial^{2}C}{\partial x^{2}}+D_{y}\frac{\partial^{2}C}{\partial y^{2}}-v_{ys}\frac{\partial C}{\partial y}$$

where *R_d_* is the retardation factor, *D_x_* is the transverse hydraulic dispersion coefficient, *D_y_* is the longitudinal hydraulic dispersion coefficient, and *v_ys_* is the longitudinal permeability velocity. All are measurable constants, and the specific parameters determined by testing are given in Table 2 Note that the initial condition includes a concentration of zero inside the model, and the boundary condition is the Dirichlet boundary condition; that is, the limited boundary concentration *C*_0_ is 150 mg/L.

### 4.2. Numerical Results

#### 4.2.1. Phenol Migration in the Soil Column

**Figure 8:**
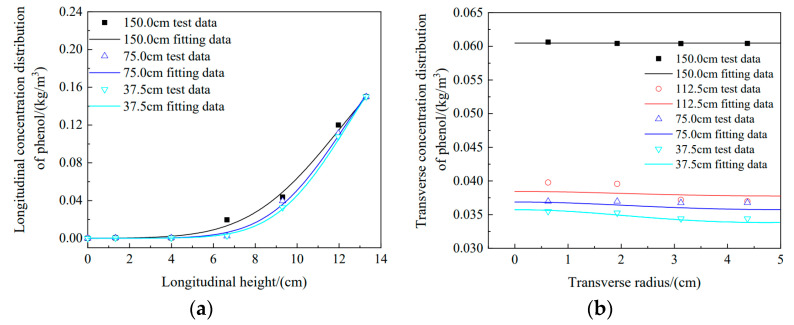
Longitudinal and transverse simulations and comparison of data for phenol concentration: (**a**) longitudinal simulation, (**b**) transverse simulation.

Figure 8a shows a comparison between the longitudinal simulation data and experimental data under longitudinal conditions, and three water head heights (150 cm, 75 cm, and 37.5 cm) were selected for the figure. The experimental data basically agreed with the simulation data, which demonstrates the accuracy of the experimental data and the feasibility of the simulation. Figure 8b shows the comparison of the transverse simulation data with experimental data for four different water head heights. As seen from the figure, the lower the water head height is, the more obvious the transverse diffusion, and the larger the fluctuation range for concentration changes. When the water head height was 37.5 cm, the transverse concentration distribution of phenol showed an obvious downward trend from 0 cm to 5 cm, and the lowest value was 0.0388 kg/m^3^. When the water head height was 150 cm, there were no obvious changes in either the simulation data or the experimental data. The experimental values for the four radii were 0.06063, 0.06043, 0.06042, and 0.6042 kg/m^3^, and the range of simulated values was approximately 0.06047 kg/m^3^. These phenomena demonstrated two things: first, the diffusion direction of pollutants in soil was mainly longitudinal with a high water head; and second, there was a small difference between the transverse experimental data and the simulated data for phenol, which showed that there was transverse migration of pollutants in the soil column.

#### 4.2.2. Migration of Rhodamine B in the Soil Column

**Figure 9:**
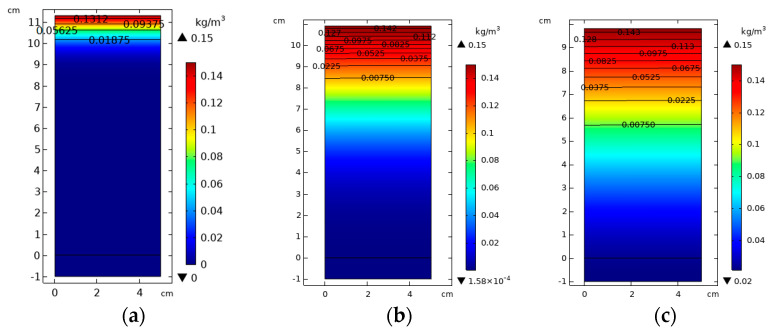
Migration of rhodamine B in soil columns: (**a**) kaolin-CTMAB bentonite, (**b**) kaolin-sodium bentonite, and (**c**) kaolin.

The migration of rhodamine B in kaolin, kaolin-sodium bentonite, and kaolin-CTMAB bentonite columns is shown from left to right in Figure 9. As with the experimental results, the simulated bottom boundary concentration was 23.3 mg/L after 67 days. The retarding effect of kaolin-sodium bentonite on rhodamine B was also small. As seen from the nephogram, the pollutant migration range was large after 67 days, and a small amount of rhodamine B solution with a concentration of 0.356 mg/L seeped out at the bottom. Under simulated conditions, rhodamine B solution did not break down the kaolin-CTMAB bentonite column, and the nephogram showed that the diffusion range of rhodamine B was small and mainly concentrated in the upper 2 cm. This is basically consistent with rhodamine B pollution, as shown in Figure 8, which showed no breakdown.

#### 4.2.3. Parameter Sensitivity Analysis

**Figure 10:**
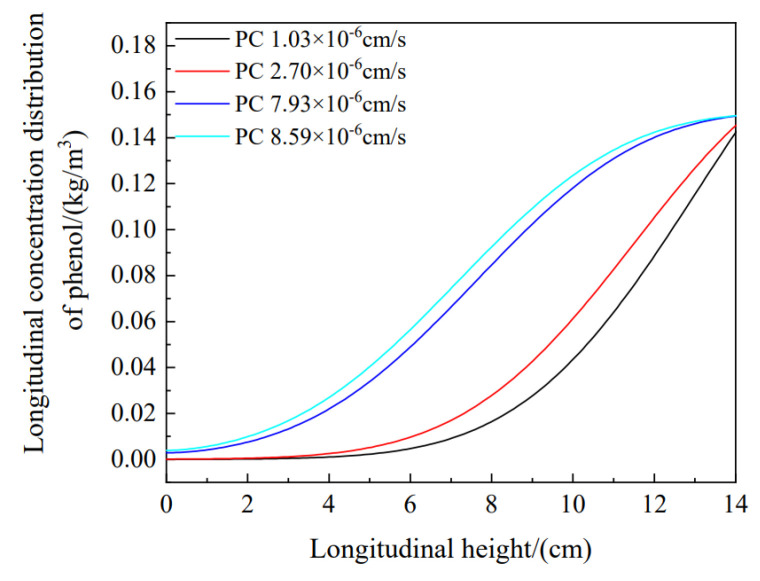
Relationships between the longitudinal distributions of pollutants and permeability coefficients (PCs).

Figure 10 shows changes in the longitudinal diffusion of pollutants with variations in the permeability coefficient, and the phenol concentrations with progressively increasing permeability coefficients are shown from bottom to top. The figure shows that the permeability coefficient was proportional to the phenol concentration in the soil column. The permeability coefficient played an important role in longitudinal migration of the pollutant. When the permeability coefficient increased, pollutant migration accelerated. When the permeability coefficient decreased, this migration slowed. Taking the same height of 8 cm as an example, the longitudinal phenol concentrations, from small to large permeability coefficients, were 0.015, 0.026, 0.082, and 0.088 kg/m^3^. It is thus proven that the permeability coefficient had a great influence on pollutant diffusion in the longitudinal direction.

**Figure 11:**
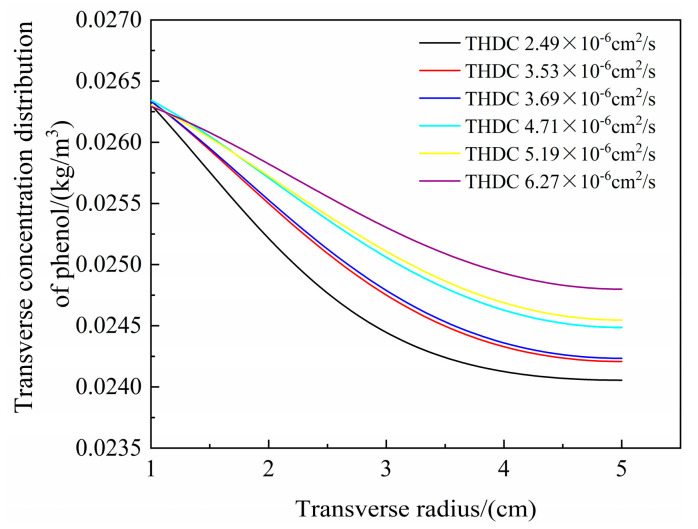
Relationship between the transverse distributions of pollutants and the hydraulic dispersion coefficients (transverse hydraulic dispersion coefficient, THDC).

Figure 11 shows the transverse distributions of pollutants with changing hydraulic dispersion coefficients. Within radii of 1–5 cm, the concentrations of pollutants in the soil column gradually decreased with increasing pollution radius. With increases in the hydraulic dispersion coefficient, transverse diffusion also increased. When the hydraulic dispersion coefficient was 6.27 × 10^−6^ cm^2^/s, the transverse migration of phenol was most effective. At a boundary of 5 cm, the phenol concentrations in order of decreasing transverse hydraulic dispersion coefficients were 0.0247, 0.0245, 0.0244, 0.0242, 0.0242, and 0.0240 kg/m^3^. A higher hydraulic dispersion coefficient resulted in faster pollutant diffusion, and the two were positively correlated. The influence of the hydraulic dispersion coefficient on the transverse concentration distribution of phenol was less than that of the permeability coefficient, indicating that pollutant migration driven by concentration was not the main method for pollutant migration in the soil column.

**Figure 12:**
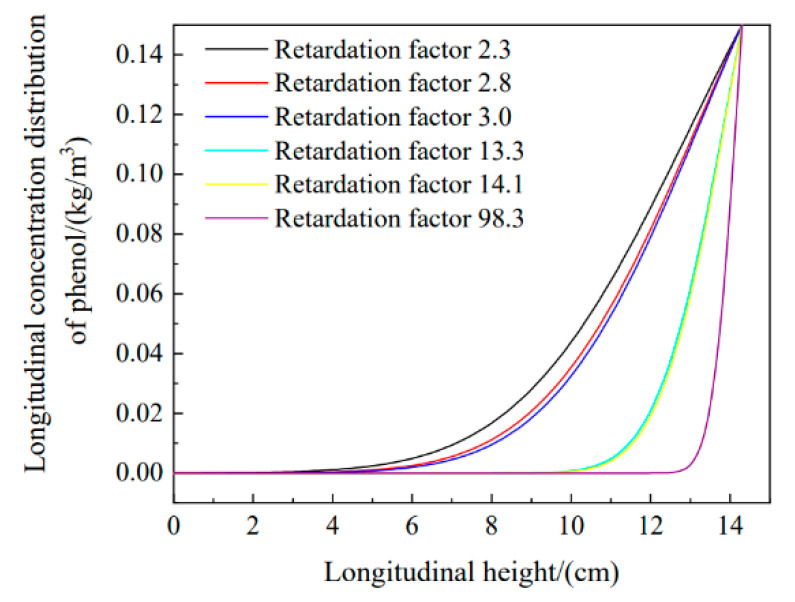
Relationship between the pollutant distribution and retardation factor.

Figure 12 shows that by changing the retardation factor when the longitudinal height was constant at 13 cm, the longitudinal concentration distribution was 0.0024, 0.058, 0.096, 0.108, and 0.112 kg/m^3^ according to the retardation factor. The retardation factor was negatively correlated with the phenol concentration, and a larger retardation factor resulted in worse pollutant diffusion and better adsorption of pollutants.

## 5. Summary of the Migration of Phenol and Rhodamine B in the Soil Column

According to the results from soil column tests and Comsol simulations based on soil column tests, phenol migration in the soil column was affected by consolidation pressure, water head height, and soil type, which was mainly reflected by the migration parameters. Consolidation pressure affected the permeability coefficient. With increasing consolidation pressure, the permeability coefficient decreased gradually, and the extent of phenol migration in the soil column subjected to a high consolidation pressure was lower than that in the soil column subjected to a low consolidation pressure. A larger permeability coefficient produced a larger distribution range for the longitudinal phenol concentration, and the permeability coefficient was directly related to the longitudinal diffusion range of pollutants.

The CTMAB bentonite content affected the effective diffusion coefficient and hydraulic dispersion coefficient, and the performance of the sodium bentonite-CTMAB bentonite test group was considerably improved compared to the other groups. With increasing CTMAB bentonite content, the effective diffusion coefficient increased gradually. The longitudinal effective diffusion coefficients corresponding to 5%, 10%, and 15% CTMAB bentonite contents were 7.18 × 10^−6^, 7.20 × 10^−6^, and 8.23 × 10^−6^ cm^2^/s, respectively. The addition of the modifier opened the internal structure of montmorillonite and provided more channels for the migration of phenol in the soil, thus increasing the effective diffusion coefficient. However, the hydraulic dispersion coefficient showed no obvious trend for variations in the CTMAB bentonite content.

In the three groups of kaolin tests, the addition of CTMAB bentonite and sodium bentonite affected the longitudinal and transverse hydraulic dispersion coefficients. Among them, the kaolin-CTMAB bentonite soil column showed the lowest longitudinal and transverse hydraulic dispersion coefficients. This was because the addition of CTMAB bentonite destroyed the polymeric structure formed by sodium bentonite and kaolin, destroyed the original pollutant migration channels, deepened the complexity of the internal space structure, affected the migration of pollutant molecules in the soil column, and led to a decrease in the hydraulic dispersion coefficient. Compared with the kaolin column, the addition of CTMAB bentonite reduced the longitudinal and transverse hydraulic dispersion coefficients from 5.66 × 10^−6^ cm^2^/s and 3.53 × 10^−6^ cm^2^/s to 3.90 × 10^−6^ cm^2^/s and 2.49 × 10^−6^ cm^2^/s, respectively. The simulation results showed that variations in the transverse hydraulic dispersion coefficient had a significant impact on the transverse migration ranges of pollutants, which showed that diffusion and dispersion played major roles in the transverse movement of this test, but these two effects had a relatively small impact on variations in the pollutant concentration.

In addition, the retardation factor was the most important factor affecting phenol migration in the soil column. According to the test standard, with increases in the CTMAB bentonite content, the retardation g factor of phenol also increased in the adsorption tests for sodium bentonite mixed with different CTMAB bentonite contents; the retardation factors were 2.3, 2.8, and 3.0, respectively, which indicated that an increase in CTMAB bentonite content enhanced adsorption by the soil. The addition of CTMAB bentonite to kaolin had a greater effect on the retardation factor for rhodamine B. In kaolin, kaolin-sodium bentonite, and kaolin-CTMAB bentonite columns, the retardation factors for rhodamine B were 14.1, 13.3, and 98.3, respectively. The retardation factor for the soil sample with CTMAB bentonite increased by 600% compared with the factor for the kaolin column without any added soil. From the simulation results, the level of rhodamine B adsorption by the kaolin-CTMAB bentonite column was also the highest. Moreover, the simulation results showed that the permeability coefficient and hydraulic dispersion coefficient were unchanged, and the diffusion of the pollutants was inversely proportional to the retardation factor. A larger retardation factor led to more obvious adsorption of pollutants, more pollutants were contained in the soil column, and the diffusion range for pollutants in the soil column was smaller.

## 6. Conclusions

In this study, soil columns were constructed to measure consolidation, diffusion, and migration breakdown to simulate pollutant migration in a soil column under different water head heights. The resulting migration parameters were imported into Comsol software, and the corresponding working conditions were simulated and compared with test data. By changing the migration parameters, we observed their influence on pollutant migration, and the following conclusions were obtained:

- With increasing consolidation pressure, the permeability coefficient decreased gradually. Sodium bentonite with 10% CTMAB showed the highest impermeability, and the permeability coefficient was 1.03 × 10^−6^ cm/s. With increasing CTMAB bentonite content, changes in the adsorbent pore ratio with higher CTMAB bentonite content exhibited a certain delay. Kaolin sodium bentonite showed good compressibility. The permeability coefficient for the soil sample with CTMAB bentonite was 3.49 × 10^—7^ cm/s, which was lower than those of kaolin and kaolin sodium bentonite and indicated good impermeability.
- The hydraulic transverse and longitudinal dispersion coefficients for phenol transported in sodium bentonite-10% CTMAB bentonite were the lowest at 4.71 × 10^−6^ cm^2^/s and 8.48 × 10^−6^ cm^2^/s, respectively. The retardation factor for the soil column with a CTMAB bentonite content of 15% was the largest at 3.0. The soil column with CTMAB bentonite showed good adsorption of rhodamine B; the retardation factor was 98.3, and the transverse and longitudinal hydraulic dispersion coefficients were the lowest at 2.49 × 10^−6^ cm^2^/s and 3.90 × 10^−6^ cm^2^/s, respectively. The test results for the indoor unit indicated that the kaolin CTMAB bentonite column adsorbed the most rhodamine B.
- Under longitudinal conditions, the simulated data for water head heights of 150 cm, 75 cm, and 37.5 cm were compared with experimental data. They all agreed well, which demonstrated the feasibility of simulating migration with Comsol software. Transverse diffusion became more evident from high to low water head heights, and the experimental data for the four water head heights were in good agreement with the simulation results. It was shown that the migration parameters measured experimentally basically described the actual migration states of pollutants in the soil column.
- The diffusion of pollutants in the soil column was tested by changing the migration parameters. The migration rule for pollutants in the soil column can be predicted with migration parameters. The higher the content of CTMAB bentonite is, the more obvious the retardation effect on the migration of organic pollutants.

## Figures and Tables

**Table 1:** Consolidation, diffusion, and migration breakdown tests of the soil column.

Soil Proportion in the Column	Consolidation Pressure (kPa)	Water Head Height (cm)	Solute (mg/L)	Test Time (Days)
Sodium bentonite-5% CTMAB bentonite	25, 50, 100, and 200	37.5, 75, 112.5, and 150	Phenol (150)	20
Sodium bentonite-10% CTMAB bentonite	25, 50, 100, and 200	37.5, 75, 112.5, and 150	Phenol (150)	20
Sodium bentonite-15% CTMAB bentonite	25, 50, 100, and 200	37.5, 75, 112.5, and 150	Phenol (150)	20
Kaolin	25, 50, 100, and 200	150	Rhodamine B (150)	10, 20, 35, 39, 41, 43, 50, and 67
Kaolin-10% sodium bentonite	25, 50, 100, and 200	150	Rhodamine B (150)	10, 20, 35, 39, 41, 43, 50, and 67
Kaolin-10% CTMAB bentonite	25, 50, 100, and 200	150	Rhodamine B (150)	10, 20, 35, 39, 41, 43, 50, and 67
Sodium bentonite-5% CTMAB bentonite	25, 50, 100, and 200	150	Chloride ion solution (150)	5
Sodium bentonite-10% CTMAB bentonite	25, 50, 100, and 200	150	Chloride ion solution (150)	5
Sodium bentonite-15% CTMAB bentonite	25, 50, 100, and 200	150	Chloride ion solution (150)	5
Kaolin	25, 50, 100, and 200	150	Chloride ion solution (150)	5
Kaolin-10% sodium bentonite	25, 50, 100, and 200	150	Chloride ion solution (150)	5
Kaolin-10% CTMAB bentonite	25, 50, 100, and 200	150	Chloride ion solution (150)	5

**Table 2:** Migration parameters.

Type	Consolidation Pressure/(kPa)	Natural Density/(g/cm^3^)	Permeability Coefficient × 10^−6^/(cm/s)	Soil Column Height/(cm)	Void Ratio	Bending Factor	Longitudinal Effective Diffusion Coefficient × 10^−6^/(cm^2^/s)	Transverse Effective Diffusion Coefficient × 10^−6^/(cm^2^/s)	Longitudinal Hydraulic Dispersion Coefficient × 10^−6^ /(cm^2^/s)	Transverse Hydraulic Dispersion Coefficient × 10^−6^ /(cm^2^/s)	Retardation Factor
Sodium bentonite-5% CTMAB bentonite	25	2.056	12.700	13.8	0.0783	—	—	—	—	—	2.3
	50	2.056	2.450	13.8	0.0741	—	—	—	—	—	2.3
	100	2.056	2.070	13.8	0.0700	—	—	—	—	—	2.3
	200	2.056	1.970	13.8	0.0466	0.7419	7.180	3.970	9.500	5.190	2.3
Sodium bentonite-10% CTMAB bentonite	25	2.027	8.590	13.3	0.0663	—	—	—	—	—	2.8
	50	2.027	7.930	13.3	0.0537	—	—	—	—	—	2.8
	100	2.027	2.700	13.3	0.0451	—	—	—	—	—	2.8
	200	2.027	1.030	13.3	0.0396	0.7440	7.200	3.990	8.480	4.710	2.8
Sodium bentonite-15% CTMAB bentonite	25	2.053	5.380	11.4	0.0671	—	—	—	—	—	3.0
	50	2.053	2.360	11.4	0.0654	—	—	—	—	—	3.0
	100	2.053	2.000	11.4	0.0596	—	—	—	—	—	3.0
	200	2.053	1.680	11.4	0.0229	0.8510	8.230	4.560	10.100	6.270	3.0
Kaolin	25	2.005	4.070	9.8	0.0826	—	—	—	—	—	14.1
	50	2.005	2.260	9.8	0.0795	—	—	—	—	—	14.1
	100	2.005	1.880	9.8	0.0762	—	—	—	—	—	14.1
	200	2.005	1.330	9.8	0.0662	0.5008	4.840	2.680	5.660	3.530	14.1
Kaolin-sodium bentonite	25	1.983	6.040	10.9	0.0727	—	—	—	—	—	13.3
	50	1.983	5.540	10.9	0.0617	—	—	—	—	—	13.3
	100	1.983	4.910	10.9	0.0481	—	—	—	—	—	13.3
	200	1.983	3.410	10.9	0.0265	0.1525	1.470	0.817	4.890	3.690	13.3
Kaolin-CTMAB bentonite	25	1.980	1.950	11.3	0.0837	—	—	—	—	—	98.3
	50	1.980	0.521	11.3	0.0829	—	—	—	—	—	98.3
	100	1.980	0.391	11.3	0.0823	—	—	—	—	—	98.3
	200	1.980	0.349	11.3	0.0751	0.2543	2.460	1.360	3.900	2.490	98.3

## Data Availability

The data that support the findings of this study are available from the corresponding author upon reasonable request.
